# Evaluation of SARS-CoV-2 infection risks after primary vaccination with BNT162b2, BBIBP-CorV, or ChAdOx1-nCOV-19 and after homologous and heterologous booster vaccinations with these vaccines and evaluation of SARS-CoV-2 reinfection profiles

**DOI:** 10.37796/2211-8039.1412

**Published:** 2023-09-01

**Authors:** Soulandi Djorwé, Amale Bousfiha, Néhémie Nzoyikorera, Joseph Nyandwi, Bellamine Kawthar, Abderrahim Malki

**Affiliations:** a Laboratory of Physiopathology and Molecular Genetics, Faculty of Sciences Ben M'Sik, Hassan II University of Casablanca (Morocco), Avenue Cdt Driss El Harti, Sidi Othman, PB 7955, Casablanca, Morocco; b Bourgogne Laboratory of Medical and Scientific Analysis, 136, Residence Belhcen, Bd Bourgogne, Casablanca, Morocco; c National Reference Laboratory, National Institute of Public Health, Burundi; d Higher Institute of Biosciences and Biotechnology, Mohammed VI University of Health Sciences (UM6SS), Casablanca, Morocco; e Laboratory of Microbial Biotechnology and Infectiology Research, Mohammed VI Center for Research & Innovation, Mohammed VI University of Health Sciences (UM6SS), Casablanca, Rabat, Morocco; f Département de Médecine, Faculté de Médecine, Université du Burundi, Burundi; g Ministére de la Santé Publique et de la Lutte Contre le Sida, Institut National de Santé Publique de Bujumbura, Burundi

**Keywords:** BBIBP-CorV, BNT162b2, ChAdOx1 nCoV-19, Reinfection, Vaccination status

## Abstract

**Background:**

The emergence of SARS-CoV-2 variants has significantly increased the number of cases of COVID-19 among vaccinated individuals, raising concerns about the effectiveness of current vaccines. The aim of this study was to analyze the SARS-CoV-2 infection risks after primary vaccination with BNT162b2, BBIBP-CorV, or ChAdOx1-nCOV-19 and after homologues and heterologous booster vaccinations with these vaccines, as well as the profiles of reinfected patients.

**Methods:**

We analyzed retrospectively 1082 patients vaccinated or unvaccinated with BNT162b2, BBIBP-CorV, and/or ChAdOx1nCoV-19 vaccines to determine their SARS-CoV2 infection statuses using the reverse transcription-polymerase chain reaction (RT-PCR) in addition to their clinical features. The infection risks of patients receiving the different vaccine regimens were compared using multivariate logistic regression analysis, comparing the adjusted OR of a positive COVID-19 test result.

**Results:**

Among 596 vaccinated patients, 53%(n = 286) tested positive for SARS-CoV-2 and 57%(n = 310) tested negative. Among positive cases, 10 were reinfection cases. The risk of SARS-CoV-2 infection was 1.6 (adj. OR) for patients who received one dose compared with those who received two doses (95% CI = 1.3–1.8; p < 0.01).The risk was 2.6 (adj. OR) for patients who received one dose compared with those who received three doses (95%CI = 2.1–3.3; p < 0.01), and 1.6 (adj. OR) for patients who received two doses compared with those who received three doses (95% CI = 1.3–2; p < 0.01). The patients who received two doses that were heterologous to that of the primary vaccine had the lowest risk of infection. Booster vaccinations (third dose) significantly reduced the number of positive cases with an acceptable safety profile. Higher cycle-threshold (Ct) values (indicative of viral load) were observed in vaccinated patients, whereas low Ct values were observed in unvaccinated patients.

**Conclusion:**

A complete cycle of vaccination with homologous vaccines or heterologous vaccines resulted in an acceptable reduction in SARS-CoV-2 infection. Further, vaccination was associated with a reduction in viral load.

## 1. Introduction

The COVID-19 pandemic has had a major global impact, causing significant morbidity and mortality worldwide. However, COVID-19 vaccines have been developed with well-documented efficacy to prevent and limit the spread of SARS-CoV-2. Clinical trials have shown that COVID-19 vaccines are safe and immunogenic, with proven efficacy against infection in randomized controlled trials (RCT) [1]. In response to this imminent threat of COVID-19, some COVID-19 vaccine manufacturing companies received emergency approval from the Food and Drug Administration (FDA) for the use of their vaccine in several countries [2]. These include the BNT162b2 messenger RNA (mRNA) vaccine (Pfizer-BioNTech) by the UK Medicines Regulatory Agency. Other COVID-19 vaccines have since been approved, including the Oxford-AstraZeneca adenoviral vector vaccine (ChAdOx1 nCOV-19) [2,3]. China and some countries, including Morocco, Egypt and Jordan, have approved the Sinopharm vaccine (BBIBPCorV, an inactivated coronavirus vaccine) [2,4]. These vaccines were modeled using different development approaches, so some of their characteristics differ, such as efficacy and storage conditions [2]. The COVID-19 pandemic has compelled health authorities around the world to develop different vaccination plans. In Morocco, as in other countries, it was decided to start vaccination mainly with high-risk groups, including the elderly and health and education personnel [5]. On January 28, 2021, a vaccination campaign was launched in Morocco. At the time of writing of this article, 2,4797,469 people have been vaccinated (partially vaccinated or fully vaccinated) [6]. However, the effectiveness of COVID-19 vaccination in preventing new SARS-CoV-2 infections in the general community is still unclear, as some individuals, although vaccinated, have still tested positive for COVID-19. Several studies have found that three doses of a SARS-CoV-2 vaccine are more effective in preventing serious consequences than two doses [7,8]. Further, the emergence of SARS-CoV-2 variants has raised concerns about the efficacy of the vaccine dosing scheme. The Omicron variant was reported to escape most neutralizing antibodies. In some studies, the efficacy of booster doses was lower for the Omicron variant than for other variants; however, booster doses were still associated with protection against SARS-CoV-2. Thus, many countries implemented booster doses with homologous and heterologous vaccines to boost the existing immune response [9].

In this study, we are interested in comparing the infection risks of patients vaccinated once with those vaccinated with booster doses of homologous and heterologous vaccines. Currently, faced with the problems of variable supply and logistical challenges of COVID-19 vaccines, booster vaccination with heterologous vaccines against COVID-19 has emerged as superior alternative for the immune protection of populations [10]. In this study, we also conducted a retrospective longitudinal study of laboratory-confirmed SARS-CoV-2 reinfection cases. This study is one of the few studies of COVID-19 reinfection at the national level, and data is scarce on the rate of reinfection in the community and on the factors that could potentially increase the risk of reinfection. It should be noted that monitoring the risk of reinfection during pathogen emergence is useful for assessing the impact of immunity conferred by the type of vaccine administered, and would contribute to the improvement of future vaccines [11]. Confirmation of reinfection requires either the use of PCR results to identify suspected reinfections ≥90 days after the initial infection or sequencing of viruses isolated from initial and later infections [12]. At the time of writing this manuscript, the Omicron (B.1.1.529) variant had become dominant in Morocco. The first case of the Omicron variant was reported on December 15, 2021. Among samples sequenced in the first week of January 2022, 60–70% were infections due to the Omicron variant [13,14].

The aim of this study was to analyze the SARS-CoV-2 infection risks after primary vaccination with BNT162b2, BBIBP-CorV, or ChAdOx1-nCOV-19 and after homologues and heterologous booster vaccinations with these vaccines. The study also reports the statuses of SARS-CoV-2 re-infection among patients who had been partially vaccinated.

## 2. Patients and methods

### 2.1. Study population and sampling

This is a retrospective analytical study that was conducted on vaccinated subjects suspected of being infected with SARS-CoV-2 and on subjects who had been in contact with confirmed COVID-19 positive subjects, as outlined in the WHO guidelines [15], as well as those who came for post-treatment control after being infected by SARS-CoV-2.

According to WHO, a SARS-CoV-2 contact is a person who has had any one of the following exposures to a probable or a confirmed case of SARS-CoV-2 infection.

The most common contact cases are:

- Face-to-face contact with a probable or confirmed case.
- Direct physical contact with a probable or confirmed case.
- Direct care for a patient with probable or confirmed COVID-19 disease without the use of recommended personal protective equipment (PPE).

On the other hand, the exposure must have occurred during the infectious period of the case, which is defined as follows:

- Exposure to a symptomatic case: 2 days before and 10 days after symptom onset of the case, plus 3 days without symptoms or 3 days with improving symptoms, for a minimum period of 13 days after symptoms onset.
- Exposure to an asymptomatic case: 2 days before and 10 days after the date on which the sample that led to confirmation was taken [15].

In this study, patients were grouped according to the number of vaccinations they received and according to whether they received vaccinations with homologous or heterologous vaccines (Table 2). In addition, among confirmed positive cases, some were reinfection cases (Table 3). This study was conducted in January 2022. The samples were obtained using nasopharyngeal swabs and were collected in tubes containing stable universal viral transport medium (Viral Transport Medium; Argene Biotechnology, Turkey) after recording clinical and demographic information.

### 2.2. RNA extraction and purification

Nextractor® NX-48SGenolution (Genolution Inc, Seoul, Korea) was used for viral RNA extraction and purification according to the manufacturer's recommendations.

### 2.3. Detection of SARS-CoV-2 RNA by RT-PCR

The presence of SARS-CoV-2 RNA was confirmed by using the Covsign amplification kit (Singuway Biotech Inc, China), which is designed to detect SARS-CoV-2 RNA by RT-PCR of specific regions of three gene targets: envelope (*E* ), nucleocapsid (*N*), and open reading frame 1 ab (*ORF1ab*). The sample was considered positive when two or three target signals were detected at a cycle threshold (Ct) < 40, as described in the manufacturer's instructions. The sensitivity of the assay was 519 copies/ml. All RTPCR reactions were run on QuantStudio™ 5 (Applied Biosystems, USA) with QuantStudio™ Design & Analysis Software version 1.5.1.

The results were interpreted according to the manufacturer's instructions.

### 2.4. COVID-19 reinfection case definition

In this study, a case of COVID-19 was defined as a reinfection if the following criteria were met:

(1) If a positive result was recorded for a patient after an interval ≥90 days after the initial laboratory-confirmed infection; (2) If the patient had at least one negative PCR result between the two infections. The interval to reinfection was calculated from the date of the first negative PCR result after the initial infection to the date of the next positive result [16].

### 2.5. Statistical analysis

Statistical analyses were performed and graphs were generated using SPSS version 23.0 (SPSS Inc., Chicago, IL, USA) and Graphad Prism 8 OS X version 8.0.1 Software, respectively. Continuous variables are presented as medians and means ± standard deviation, and interquartile ranges were calculated for normal and skewed data distributions. Comparisons between different categorical variables are presented as numbers and percentages and evaluated by Fisher's exact test or chi-squared test. The difference in (cycle threshold) Ct values between the targets was analyzed using Student's t-test. To analyze the strength of the association between risk factors for infection (SARS-CoV-2) in vaccinated and unvaccinated patients, univariate logistic regression was used to determine the odds ratio (OR), while multivariate logistic regression was used to determine the adjusted odds ratio (adj. OR). To adjust for possible confounding factors, the final model included: age, gender, symptoms, reinfection, reasons for screening, and dose received. A 95% confidence interval (95% CI) was used to indicate statistical significance. P-values (p) < 0.05 were considered statistically significant, and those <0.01 were considered highly significant.

## 3. Results

**Fig. 1:**
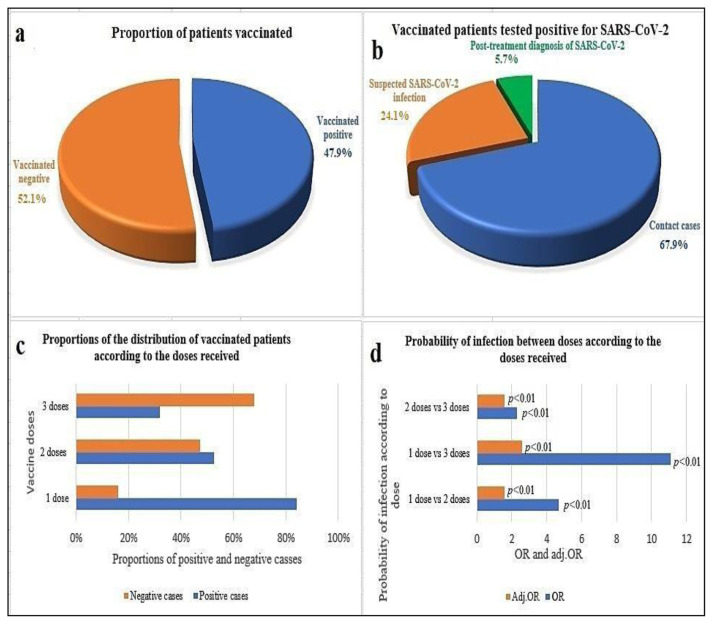
**a)** Proportions of vaccinated patients who tested positive or negative for SARS-CoV-2. **b)** Proportions of patients vaccinated for different diagnostic reasons (suspicion of COVID-19, contact with a subject positive for SARS-CoV-2, and post-treatment control after SARS-CoV-2 infection). **c**) Distribution of vaccinated subjects according to number of doses administered (one dose, two doses (homologous and heterologous) or three doses (homologous and heterologous), and infection status. **d**) Comparison between number of doses received and risk of infection (OR and adjusted OR).

This study included 1082 patients with ages ranging from 2 to 101 years. The mean age ± standard deviation was 41.8 ± 18.1 years. Among the 1082 patients, 55.1% (n = 596) had been vaccinated with at least one dose of a COVID-19 vaccine (BNT162b2, ChAdOx1 nCoV-19, or BBIBPCorV). Among vaccinated patients, 47.9% (n = 286) tested positive with an age range of 13–80 years (mean age ± standard deviation, 40.3 ± 15.7 years). By contrast, 52.1% (n = 310) of vaccinated patients aged 13–88 years tested negative (the mean age ± standard deviation was 39.8 ± 16.4 years) (Fig. 1a). Among the 47.9% (n = 286) of vaccinated patients who tested positive, 69.2% (n = 198) had been in contact with a confirmed positive case, 24.1% (n = 71) had a suspected COVID-19 infection, and 5.7% (n = 17) were tested as post-treatment controls (patients who came back for a PCR check after having been treated for a positive SARS-CoV2 infection) (Fig. 1b). According to the statistical distribution of vaccinated patients, 35.5% (n = 212) received three doses of a vaccine against SARS-CoV-2 and among these, 32.1% (n = 68) were positive cases and 67.9% (n = 144) were negative cases (p < 0.01). Fifty-six percent (n = 334) of patients received two doses of a vaccine (second shots), of which 52.7% (n = 176) tested positive and 47.3% (n = 158) tested negative (p = 0.32). 8.4% (n = 50) of patients received one dose of a vaccine, of which 84% (n = 42) tested positive and 16% (n = 8) tested negative (p < 0.01). In this study, Fisher's exact test showed that there was a significant relationship (p < 0.01) between patients who received two homologous vaccine doses versus those who received two heterologous vaccine doses and, further, between those who received three homologous vaccine doses versus those who received three heterologous vaccine doses (third shot). In our analysis, unvaccinated subjects had a 1.4 higher risk of testing positive for COVID-19 than vaccinated subjects (OR = 1.4; 95% confidence interval [CI] = 0.9–1.5). The associations between SARS-CoV-2 infection and the number of doses administered (i.e., total number of patients receiving one dose, two doses, or three doses) were analyzed using the multivariate logistic regression model to obtain the odds ratio of the groups and the adjusted odds ratios (adj. OR) (Table 1).

The results showed that the risk of SARS-CoV-2 infection varied significantly (p < 0.01) according to the number of doses received (Fig. 1c-d and Table 1). The risk of infection in subjects who received one dose of vaccine compared with those who received two doses was 4.7 (OR = 4.7; 95% CI = 2.14–10.44), and the adjusted OR was 1.6 (95% CI = 1.3–1.8). This risk was 11.1 in patients who received one dose compared with those who received three doses (OR = 11.1; 95% CI = 4.94–24.97), and the adjusted OR was 2.6 (95% CI = 2.3–3.3). By contrast, the risk was 2.3 in patients who received two doses compared with those who received three doses (OR = 2.3; 95% CI = 1.64–3.37), and the adjusted OR was 1.6 (95% CI=1.3–2) (Table 1). The unvaccinated patient group was used as a reference group in the logistic regression analysis (Table 2; Fig. 1 c-d). Subjects who received two doses of different vaccines (heterologous vaccines: (BNT162b2 + ChAdOx1 nCoV-19), (BNT162b2 + BBIBP-CorV), or (ChAdOx1 nCoV-19+BBIBP-CorV)) had a lower risk of testing positive than those who received two doses of the same vaccine (homologous vaccine group) (Table 2). Patients who received three doses of homologous vaccines or three doses of heterologous vaccines had a very low risk of testing positive. However, despite the fact that vaccination with heterologous vaccines was effective, the number of patients who received three doses of heterologous vaccines (two doses BNT162b2 + one dose ChAdOx1 nCoV-19; two doses BNT162b2 + one dose BBIBP-CorV; two doses ChAdOx1 nCoV-19+one dose BBIBP-CorV; 2 doses BBIBPCorV+one dose ChAdOx1 nCoV-19) was small (Table 2).

**Fig. 2:**
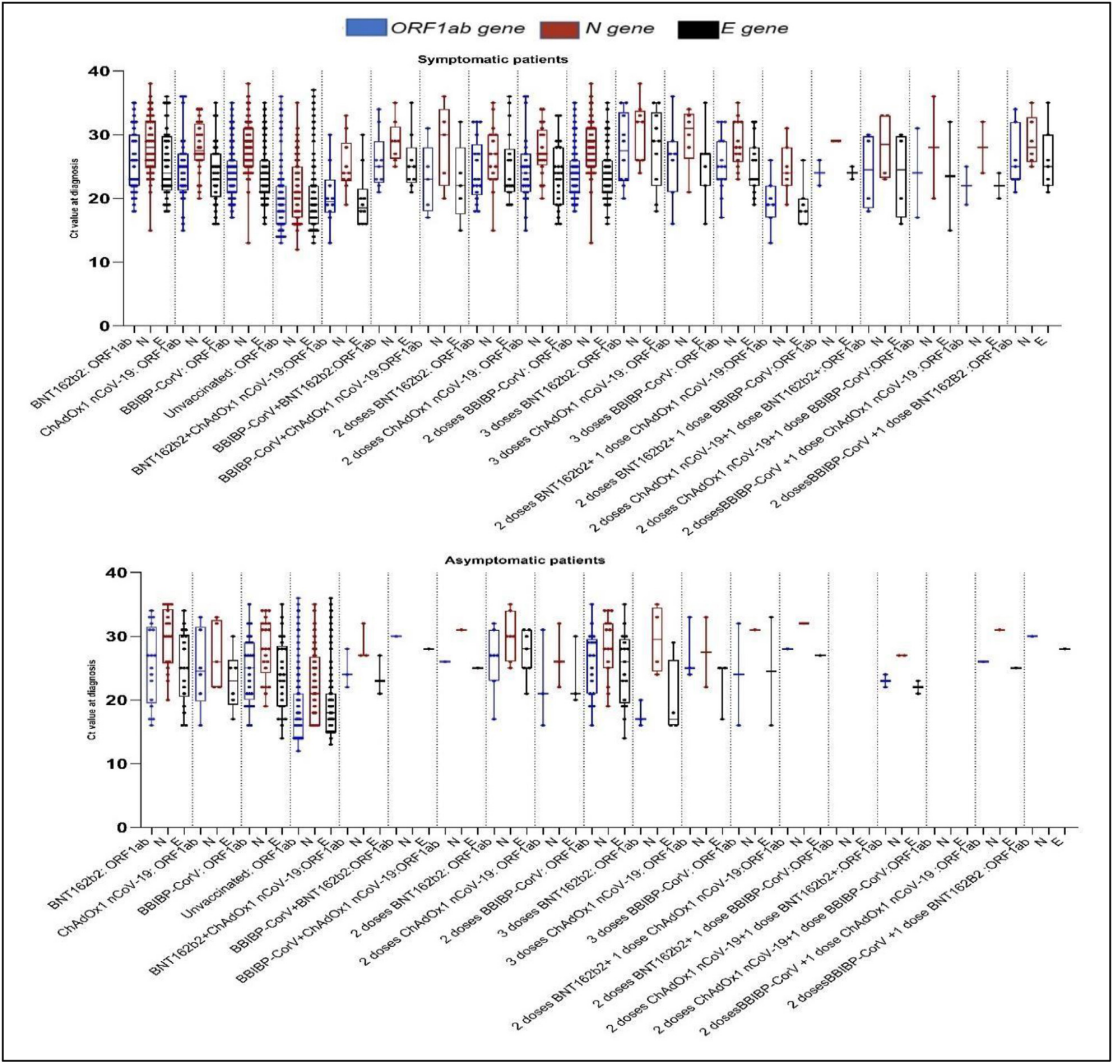
Distribution of Ct values according to type of vaccine and number of doses administered and distribution of Ct values according to the vaccination regimen among symptomatic and asymptomatic individuals. Box plots show the dispersion and frequency density of virus target genes observed in positive patients. The center lines of the whisker boxes represent median Ct values; the limits of the box plots indicate the first and third quartiles. P-values (** <0.01; *<0.05; ns: not significant). Ct values are indicative of viral load. Lee and al., 2021 described the details of equivalent viral loads in copies per milliliter according to the formula:(log10 viral load = 12.0–0.38x Ct) [17,18].

In this study, we used the Ct value, which is a semi-quantitative estimate of viral load, to compare viral loads of vaccinated and unvaccinated subjects (Fig. 2). The differences in Ct values between target genes were analyzed using Student's t test. However, the difference between the Ct values of the same target genes were all statistically significant (p < 0.01). In vaccinated, infected index patients, the Ct values were generally high. Among all patients who tested positive for COVID-19, 71% had symptomatic infections (n = 384) whereas 29% (n = 156) had asymptomatic infections. However, in vaccinated patients, 80% (n = 231) of patients had symptomatic infections whereas 20% (n = 55) had asymptomatic infections. In unvaccinated patients, 60% (n = 153) had symptomatic infections whereas 40% (n = 101) had asymptomatic infections.

The results of the analyses were compared between symptomatic and asymptomatic patients, who were grouped according to the number of doses administered and the type of vaccine and Ct values. In the unvaccinated group, the difference between symptomatic and asymptomatic patients was statistically significant (p < 0.01); the symptomatic index patients in this group had the following median Ct and interquartile range (IQR) values: Ct *ORF1ab* = 13.5 (12–19), Ct N = 16 (13–20) and Ct E = 14 (12–19), while the asymptomatic index patients had the following values: Ct *ORF1ab* = 16 (13–20), Ct *N* = 18 (16–22), Ct *E* = 17(13–20).

The Ct values of symptomatic and asymptomatic patients who received one dose of BNT162b2 were significantly different (p = 0.01); the symptomatic index patients in this group had the following median Ct and IQR values: Ct *ORF1ab* = 23 (21–30), Ct *N* = 27 (24–33), and Ct *E* = 23 (21–30), while in the asymptomatic index patients, these values were: Ct *ORF1ab* = 27 (23–31), Ct *N* = 31 (26–35), and Ct *E* = 26.5 (21.7–31). Among patients who received one dose of ChAdOx1 nCoV-19, symptomatic index patients had the following median Ct and IQR values: Ct *ORF1ab* = 25 (21–27), Ct *N* = 27.5(26–31.7), and Ct E = 24(20–27), while in the asymptomatic index patients, these values were: Ct *ORF1ab* = 24.5 (21.7–32), Ct *N* = 31 (24–32.5), and Ct *E* = 25 (22.5–31.2). This difference was significant (p < 0.01). Among patients who received one dose of BBIBP-Cor (p < 0.01), the median Ct and IQR values of symptomatic index patients were: *ORF1ab* = 24 (21–26), Ct *N* = 28(25–31), and Ct *E* = 23 (21–26), while in the asymptomatic index patients, these values were: Ct *ORF1ab* = 27(21–29,2), Ct *N* = 28 (25–32), and Ct *E* = 24,5 (19,7–28,2) (Fig. 2). Among patients who received two doses of heterologous vaccines (BBIBP-CorV + one dose of BNT162b2), the median Ct and IQR values of symptomatic index patients were: Ct *ORF1ab* = 25(22–32), Ct *N* = 29(25–35), and Ct *E* = 25(22–30), while in asymptomatic index patients these values were: Ct *ORF1ab* = 27(median) and Ct *N* = 28 (median). This difference was significant (p = 0.01). By contrast, the median Ct and IQR values of symptomatic index patients who received two doses of a homologous vaccine (BNT162b2) were: Ct *ORF1ab* = 23(21–27, Ct *N* = 27(23–30), and Ct *E* = 22(21–27), while in asymptomatic index patients these values were: Ct *ORF1ab* = 27(17–32), Ct *N* = 30(25–35), and Ct *E* = 28(21–31)). The difference was statistically significant (p < 0.02). The median Ct and IQR values of symptomatic index patients receiving two doses of ChAdOx1 nCoV-19 were: Ct *ORF1ab* = 24(22–26), Ct *N* = 27(26–30), and Ct *E* = 24(20–25), while in asymptomatic index patients, these values were: Ct *ORF1ab* = 21(16–31), Ct *N* = 26(22–32), and Ct *E* = 21(20–30). The difference was statistically significant (p < 0.01). The median Ct and IQR values of symptomatic index patients receiving two doses of BBIBP-CorV were: Ct *ORF1ab* = 24(22–25), Ct *N* = 28(26–29), and Ct *E* = 23(22–24), while in asymptomatic index patients, these values were: Ct *ORF1ab* = 27(21–29), Ct *N* = 28(25–32). and Ct *E* = 26(20–29) (Fig. 2). The difference was statistically significant (p < 0.01).

The median Ct and IQR values of symptomatic patients who received three doses of BBIBPCorV were: Ct *ORF1ab* = 25 (23–29), Ct *N* = 28 (25–32), and Ct *E* = 23 (22–28), while in asymptomatic index patients, these values were: (Ct *ORF1ab* = 24 (16–32), Ct *N* = 31(median), and Ct *E* = 24 (16–33). The difference was statistically significant (p < 0.01). By contrast, the median Ct and IQR values of symptomatic index patients who received a third heterologous vaccine dose (two doses of BNT162b2+one dose of ChAdOx1 nCoV-19) were: Ct *ORF1ab* = 19 (13–26), Ct *N* = 24 (19–31), and Ct *E* = 18 (16–26), while in asymptomatic index patients, these values were: (Ct *ORF1ab* = 28 (median), Ct *N* = 32 (median), and Ct *E* = 27 (median). The difference was significant (p = 0.03). The median Ct and IQR values of symptomatic index patients who received a third dose with another heterologous vaccine (two doses of BBIBPCorV + one dose of BNT162b2) were: Ct *ORF1ab* = 25 (21–34), Ct *N* = 28 (25–35), and Ct *E* = 25 (21–35), while in asymptomatic index patients, these values were: (Ct *ORF1ab* = 30 (median) and Ct *E* = 28 (median) (Fig. 2). The difference was statistically significant (p = 0.03).

**Fig. 3:**
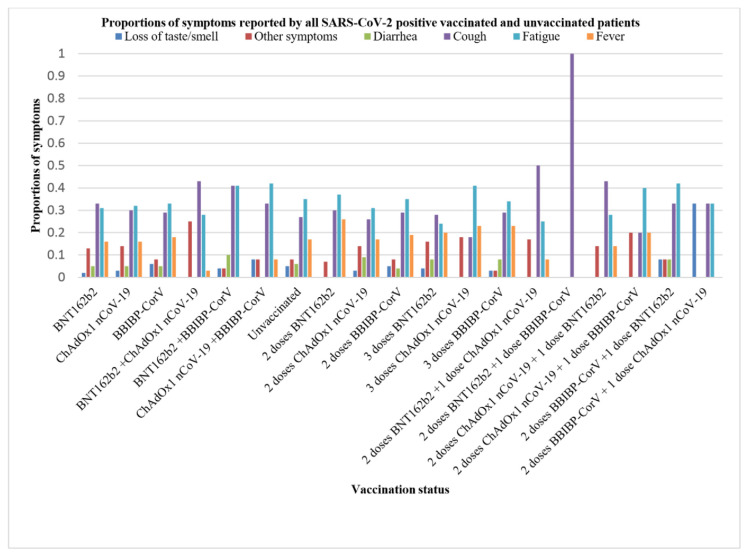
Symptoms and their proportions according to vaccination statuses.

The symptoms frequently reported by patients are shown in Fig. 3 with an OR>1. Vaccinated and unvaccinated patients presented with nearly the same proportion of symptoms. All patients developed mild symptoms. The most reported symptoms were fatigue, cough, and fever, except for patients who received a third dose of a heterologous vaccine (two doses of BNT162b2 + one dose of BBIBP-CorV) or (two doses of BBIBP-CorV + one dose of ChAdOx1 nCoV-19), among which there were fewer positive cases and therefore fewer reported symptoms.

Vaccinated-SARS-CoV-2 positive patients who had followed the SARS-CoV-2 infection treatment protocol had a negative RT-PCR test result within a median of 7 days after treatment with an IQR of 6–10 days, while unvaccinated SARS-CoV-2 positive patients had a negative RT-PCR test result within a median of 11 days with an IQR of 10–14 days (reference data are provided in table S1 in Supplementary Materials).

### 3.1. COVID-19 reinfection

Ten cases of reinfection were reported among the 596 confirmed positive cases. The mean age of the reinfected patients was 37.6 ± 17.4 years. No history of comorbidity was reported, and all patients had moderate symptoms (Table 3). Evaluation of the association between partial vaccination and SARS-CoV-2 reinfection showed no significant difference compared with nonvaccinated cases. In this study, reinfection was confirmed by RT-PCR in 10 patients with ages ranging from 16 to 70 years, with a shift of reinfection ≥4 months (Table 3). There was strong statistical evidence that reinfected cases had a longer time interval between the first and second episode of infection (mean, 290 days; 95% CI [213–367]).

**Fig. 4:**
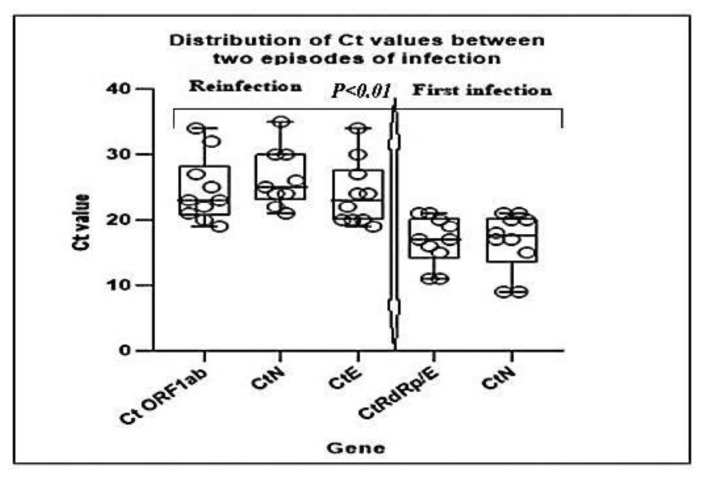
Distribution of Ct values during initial infection and second episodes of infection.

At the time of the first infection, the mean ± standard deviation Ct values of the *RdRP/E* and *N* genes were: 16.8 ± 3.6 and 16.7 ± 4.4, respectively, with respective median IQR values of 17 (14.5–20.2) and 17.5 (13.5–20.2). At the time of the second infection, the mean ± standard deviation and median (IQR) Ct values of the *ORF1ab*, *N*, and *E* genes were: 24.6 ± 5, 26.3 ± 4.5, and 24 ± 4.9 with respective median values (IQR) of 23 (20.7–28.2), 25 (23–30), and 23 (20–27.7). T-test analysis of the mean Ct values between the two infection episodes showed that the mean Ct values of the initial infection were lower (high viral load) than those of the second infection episode (low viral load) (p < 0.01) (Fig. 4). The specific Ct values of the first and second infection are shown in table S2 in Supplementary Materials.

In general, there was a significant difference in viral load between the first episode of infection and second episode of infection (Fig. 4). Patients who were partially vaccinated or unvaccinated and those previously infected had a 1.2 risk of being reinfected [OR = 1.2; 95%CI (0.28–5.93)].

## 4. Discussion

Vaccination against SARS-CoV-2 is one of the most effective measures that healthcare systems can take to reduce the infection rate and severity, and the mortality associated with the SARS-CoV-2 pandemic [7,19,20]. SARS-CoV-2 vaccination strengthens the immune system and contributes to herd immunity, which has been important for overcoming the COVID-19 pandemic [21]. In this study, we found that BNT162b2, ChAdOx1 nCoV-19, and BBIBP-CorV vaccines were effective against SARS-CoV-2 infections. Nevertheless, notable differences in the risks of infection were observed between vaccinated patients. This difference could be due to differences in the components and mechanism of action of each vaccine. Although vaccines have been shown to be effective against SARS-CoV-2, a small proportion of people still tested positive for COVID-19 after vaccination [22]. In this study, unvaccinated patients had a 1.4 fold higher risk of testing positive for COVID-19 than vaccinated patients. In addition, the risk of being infected by SARS-CoV-2 after vaccination with a booster dose was significantly lower than that without a booster dose (p < 0.01) (Table 1).

In some studies, single-dose vaccination was reported to be much less effective in preventing infection against coronavirus disease than vaccination with booster doses. The first dose triggers the immune system, while the second dose triggers the dominant immune response [23]. In accordance with our data, most of the patients who received a single dose of one of the three vaccine types tested positive for COVID-19; this would explain in part why a single dose of the SARS-CoV-2 vaccine is less effective in preventing infection [24]. In our study, patients who received one dose or two doses (second homologous dose) of the BBIBP-CorV vaccine had a significantly higher risk of COVID-19 infection than those vaccinated with BNT162b2 or ChAdOx1 nCoV-19 (Table 2). Our observations are similar to those of Boshra and colleagues who reported that the BBIBPCorV vaccine has weak immunological activity due to a significantly higher rate of COVID-19 infection among patients after receiving one dose and two homologous doses (second shot) of BBIBP-CorV; therefore, a booster dose (third dose) is necessary to activate immunological memory in patients who receive the BBIBP-CorV vaccine [25]. Many studies have reported that vaccination with heterologous vaccines (second shot) induces a stronger immune response than vaccination with homologous vaccines [10,26–28]. We found that patients who received two doses (second shot) of heterologous vaccines (Table 2) had a lower risk of testing positive than those who received two doses of homologous vaccines. According to our results, heterologous vaccination (two doses) with vaccines such as BNT162b2+ChAdOx1 nCoV-19, BBIBP-CorV + BNT162b2, and BBIBPCorV + ChAdOx1 nCoV-19 are safe, and considerably reduce the risk of infection with SARS-CoV-2.

In this study, we found that, in contrast to the second dose (second shot), the third booster dose the dose received 6 months after the second dose) provided increased protection against SARS-CoV-2 infection, supporting the idea that it establishes immunological memory [20,29]. Previous studies have reported that the antibody levels after three booster vaccinations (third shots) with homologous or heterologous vaccines, such as BBIBP-CorV or mRNA vaccines (e.g., BNT162b2), and ChAdOx1 nCoV-19 were effective at providing protection from infection and significantly enhanced long-term humoral responses against SARS-CoV-2 [26,30]. This suggested that the third dose strongly stimulates the immune system. However, some studies have reported that, three booster heterologous vaccinations (third shot), such as with the ChAdOx1 nCoV-19 vaccine and mRNA vaccine BNT162b2, generate a more robust immune responses against SARS-CoV-2 than homologous vaccination with ChAdOx1 nCoV19 vaccine by increasing the numbers of SARS-CoV-2 specific T and B cells via the stimulation of spike protein and receptor binding domain (RBD)-specific memory B cells [31,32]. In addition, vaccination with heterologous vaccines is known to better counteract SARS-CoV-2 infection than vaccination with homologous vaccines and could provide a powerful strategy for counteracting the emergence of new SARS-CoV-2 variants carrying mutations that escape the immune system [26,33,34]. In our study, we found that subjects who received the third dose, whether with a homologous or a heterologous vaccine, were less likely to test positive for COVID-19. However, the number of COVID-19-positive patients was lower after heterologous vaccination (both in patients who received a second dose of heterologous vaccination and in patients who received a booster shot of heterologous vaccination) than after homologous vaccination. Indeed, vaccination with a third dose of a homologous vaccine (BNT162b2, ChAdOx1 nCoV-19, or BBIBP-CorV) also resulted in a statistically significant reduction in the number of cases (Table 2). Nevertheless, homologous vaccination appears to show its efficacy in generating a stronger immunogenic response only when it is administered after the second dose. Furthermore, the immunogenicity of a third dose of heterologous vaccines is increasingly becoming the focus of the global fight against SARS-CoV-2. However, a wider study should be conducted on heterologous vaccination, such as heterologous vaccination with ChAdOx1 nCoV-19 + BBIBPCorV, to determine its efficacy against different variants, which would lead to a better understanding of the immunogenicity of heterologous vaccination [26]. In general, our data support further studies into the applicability of heterologous vaccination strategies against SARS-CoV-2 infection. In addition, we found that vaccination was crucial for reducing viral loads (Fig. 2). This observation is in agreement with that of Levine-Tiefenbrun and colleagues, as well as with that of Eyre and colleagues, who showed that COVID-19 vaccination reduces viral load, infectivity, and virus spread [18,35]. Studies such as that of Lee and colleagues have reported that the number of positive contact cases increases with viral load [17]. Consequently, the infectivity of SARS-CoV-2 increases with increasing viral load. In accordance with this, our study showed that 69.2% (n = 198) of SARS-CoV-2 positive, vaccinated patients had been in contact with confirmed positive cases. Our results also confirm the findings of previous studies reporting that contact cases are associated with higher transmission rates [17,18]. Furthermore, in our study, vaccination was associated with moderate Ct values (moderate viral loads). Higher Ct values (median Ct ≥ 23) were observed in vaccinated patients, whereas low Ct values (median Ct ≤ 16) were observed in unvaccinated patients (Fig. 2), which is in agreement with the results of Eyre and colleagues [18]. Interestingly, vaccinated, SARS-CoV-2 positive patients recovered 7 days (median) after treatment, in contrast to unvaccinated, SARS-CoV-2 positive patients who recovered after 11 days (median).

In this study, cases of reinfection were observed in the patient population. However, cases of reinfection coincided with the peak period of the omicron variant wave in Morocco [14]. As reported in the study of Jain and colleagues, several etiological factors may be risk factors for reinfection with COVID-19. Reinfection may be associated with low antibody titers during the initial infection and/or a reduced duration of immune prevention [36]. SARS-CoV-2 variants of concern that evade vaccine-induced immunity may also cause reinfection, which is consistent with the previous identification of immune evasion mutations [36]. Nevertheless, in our study, as in that of Bongiovanni and colleagues, the reinfected patients mostly developed milder symptoms [37]. Unlike in the study of Salehi-Vaziri and colleagues, the cases of reinfection in our study were less severe [38]. Furthermore, we are confident that the cases reported in our study were true cases of SARS-CoV-2 reinfection, because they exhibited the largest time interval between two episodes (144–414 days, respectively) (Table 3) [39].

In addition, previously infected unvaccinated patients and partially vaccinated patients had a 1.2-fold higher risk of reinfection (OR = 1.2; 95% CI = 0.28–5.93), suggesting that partial vaccination or previous infection was not significantly associated with protection against reinfection. In this study, we found a significant difference in Ct values between the initial infection and the second infection (Fig. 4). In addition, Ct values were lower during primary infection (high viral load) than during the second episode of infection.

Our study has several limitations. First, the time intervals between vaccinations were not taken into account. Furthermore, no genomic analysis of the viral strains was performed to confirm the dominant circulating variant; therefore, the efficacy of the three vaccine types against SARS-CoV-2 variants could not be determined. Also, our data were insufficient to determine whether there was a difference in efficacy between heterologous vaccination with the ChAdOx1 nCoV-19 vaccine and BBIBP-CorV vaccine. Further analysis will be required to determine the optimal combination of primary vaccine and booster vaccines for heterologous vaccination [40]. Reinfection was not confirmed by sequencing and viral culture, which are important for confirming reinfection. The retrospective nature of reinfection in this study means that the results cannot be used to infer causality of reinfection; additional prospective studies will be needed to support and complement our results.

## 5. Conclusion

All three vaccines (BNT162b2, BBIBP-CorV, and ChAdOx1 nCoV-19) were found to be safe in preventing and minimizing SARS-CoV-2 infections in this study. Further, vaccination was crucial for reducing viral load. A second dose of vaccine with a heterologous protocol of vaccination provided greater protection than that with a homologous vaccine. A third booster vaccination with a heterologous vaccine provided greater protection than that with a homologous vaccine. However, the prevalence of positive cases was rather low irrespective of whether booster vaccinations were performed with heterologous or homologous vaccines. Nevertheless, homologous vaccination appears to show its efficacy in generating a stronger immunogenic response only when it is administered after the second dose. Considering the supply problems of COVID-19 vaccines in some countries, vaccination with heterologous vaccines should be considered as an alternative for achieving herd immunity. Breakthrough reinfections in partially vaccinated and unvaccinated patients were observed, with significant differences in viral load between the two episodes of infection.

## Supplementary Data

**Table s1:** Control after treatment.

Gender	Age (year)	Result	Ct *ORF 1ab*	Ct *N*	Ct *E*	Reinfection	Loss of taste and	Other symptom	Diarrhea	Cough	Fatigue	Fever	Reasons for the diagnosis	Dose BBIBP-CorV	Dose BNT162b2	Dose ChAdOx1 nCoV-19	Control after treatment (days)
F	8	0				0	0	0	0	0	0	0	Control after treatment				15days
M	9	0				0	0	0	0	0	0	0	Control after treatment				7days
M	11	0				0	0	0	0	0	0	0	Control after treatment				10days
M	12	0				0	0	0	0	0	0	0	Control after treatment				11days
F	15	1	31	35	22	0	0	0	0	0	0	0	Contact case		1		5days
F	15	0				0	0	0	0	0	0	0	Contact case		2		7days
M	15	0				0	0	0	0	0	0	0	Contact case				16days
M	16	0				0	0	0	0	0	0	0	Contact case				9days
F	17	1	34	35	30	0	0	0	0	0	0	0	Contact case		1		11days
M	17	0				0	0	0	0	0	0	0	Contact case				15days
F	18	0				0	0	0	0	0	0	0	Contact case	2			6days
M	18	1	33	35	32	0	0	0	0	0	0	0	Contact case		1		6days
M	19	0				0	0	0	0	0	0	0	Contact case		1		7days
M	19	0				0	0	0	0	0	0	0	Contact case				8days
F	20	0				0	0	0	0	0	0	0	Contact case	2			6days
F	21	0				0	0	0	0	0	0	0	Contact case	1			5days
F	21	1	21	27	21	0	0	0	0	0	0	0	Contact case	1			13days
F	22	0				0	0	0	0	0	0	0	Contact case	2			7days
F	24	0				0	0	1	0	0	0	0	Contact case	2			7days
F	25	1	31	34	31	0	0	0	0	0	0	0	Contact case		2		7days
F	25	0				0	0	0	0	0	0	0	Contact case	2			10days
F	25	0				0	0	0	0	0	0	0	Contact case		2		7days
F	26	0				0	0	0	0	0	0	0	Contact case				7days
F	26	0				0	0	0	0	0	1	0	Control after treatment				7days
F	27	0				0	0	0	0	0	0	0	Contact case	2			5days
F	27	0				0	0	0	0	0	0	0	SARS-CoV-2 suspicion	2			5days
F	28	0				0	0	0	0	0	0	0	Contact case	1			7days
F	29	0				0	0	0	0	0	0	0	Control after treatment				15days
F	29	1	20	24	20	0	0	0	0	0	0	0	Contact case				21days
M	29	0				0	0	0	0	0	0	0	Contact case	2			7days
F	29	0				0	0	0	0	0	0	0	SARS-CoV-2 suspicion	1			10days
M	29	0				0	0	0	0	0	0	0	SARS-CoV-2 suspicion				14days
F	31	0				0	0	0	0	0	0	0	Control after treatment				5days
F	32	0				0	0	0	0	0	0	0	Control after treatment				15days
F	32	0				0	0	0	0	0	0	0	Control after treatment	2			5days
M	32	0				0	0	0	0	0	0	0	Control after treatment				11days
M	32	0				0	0	0	0	0	0	0	Control after treatment				13days
F	32	0				0	0	0	0	0	0	0	Control after treatment		2		9days
F	32	0				0	0	1	0	0	0	0	Contact case				5days
M	32	0				0	0	0	0	0	0	0	Control after treatment				14days
F	33	1	9	12	9	0	0	0	0	0	0	0	Control after treatment				5days
F	33	1	30	34	29	0	0	0	0	0	0	0	Control after treatment	2			5days
F	34	0				0	0	0	0	0	0	0	Control after treatment				15days
M	35	1	20	23	17	0	0	0	0	0	0	0	Control after treatment				13days
M	35	0				0	0	0	0	0	0	0	Control after treatment	2			7days
M	36	0				0	0	0	0	0	0	0	Control after treatment	1			6days
F	36	1	16	18	17	0	0	0	0	0	0	0	Control after treatment				15days
F	36	0				0	0	0	0	0	0	0	Control after treatment	3			5days
F	36	0				0	0	0	0	0	0	0	Control after treatment	2			11days
F	36	0				0	0	0	0	0	0	0	Control after treatment				11days
F	37	0				0	0	0	0	0	0	0	Control after treatment	1			10days
M	37	1	16	18	19	0	0	0	0	0	0	0	Control after treatment				6days
M	37	0				0	0	0	0	0	0	0	Control after treatment			2	7days
M	38	0				0	0	0	0	0	0	0	Control after treatment	2			15days
F	38	0				0	0	0	0	0	0	0	Control after treatment	1			5days
M	38	0				0	0	0	0	0	0	0	Control after treatment				10days
M	39	0				0	0	0	0	0	0	0	SARS-CoV-2 suspicion	2			9days
F	39	0				0	0	0	0	0	0	0	Control after treatment			2	7days
M	39	0				0	0	0	0	0	0	0	Control after treatment				15days
M	39	0				0	0	0	0	0	0	0	SARS-CoV-2 suspicion				7days
F	39	0				0	0	0	0	0	1	0	Control after treatment	2		1	7days
F	39	0				0	0	0	0	0	0	0	Control after treatment	1			7days
M	40	1	34		34	0	0	1	0	1	1	1	Control after treatment	2			6days
F	41	0				0	0	0	0	0	0	0	Control after treatment	1			11days
F	42	0				0	0	0	0	0	0	0	Control after treatment	2			7days
F	42	0				0	0	0	0	1	0	0	Control after treatment	2			9days
M	42	0				0	0	0	0	0	0	0	Control after treatment	2			8days
M	43	1	30	34	30	0	0	0	0	0	0	0	Control after treatment	2			7days
F	44	0				0	0	0	0	0	0	0	Control after treatment	2			6days
M	45	0				0	0	0	0	0	0	0	Control after treatment	3			11days
F	45	1	27	31	26	0	0	0	0	0	0	0	Control after treatment	2			4days
F	45	0				0	0	0	0	0	0	0	Control after treatment				13days
F	45	0				0	1	0	0	1	1	0	Control after treatment	3			5days
M	45	0				0	0	0	0	0	0	0	Control after treatment				15days
F	46	0	34	40	36	0	0	0	0	1	0	0	Control after treatment				10days
F	46	0				0	0	0	0	0	0	0	Control after treatment	2	1		5days
F	46	0				0	0	1	0	0	0	0	Control after treatment	2			7days
F	46	0				0	0	0	0	0	0	0	Control after treatment	3			9days
F	47	0				0	0	1	0	0	0	0	SARS-CoV-2 suspicion		2		10days
F	47	0				0	0	0	0	0	0	0	Control after treatment	3			10days
M	47	0				0	0	0	0	0	0	0	Control after treatment				12days
F	47	0				0	1	0	0	0	1	0	Control after treatment	2			7days
F	48	0				0	0	0	0	0	0	0	Control after treatment	2			5days
F	49	0				0	0	0	0	0	0	0	Control after treatment		3		10days
M	49	0				0	0	0	0	0	0	0	SARS-CoV-2 suspicion	2			7days
F	50	0				0	0	0	0	0	0	0	Control after treatment				10days
M	51	0				0	0	0	0	0	0	0	Control after treatment				15days
M	52	1	28	32	27	0	0	0	0	0	0	0	Control after treatment		2	1	8days
F	52	0				0	0	0	0	0	0	0	Control after treatment				15days
F	52	0				0	0	0	0	0	0	0	Control after treatment			2	10days
F	53	1	35		34	0	0	0	0	0	1	0	Control after treatment	2			6days
F	54	0				0	0	0	0	0	0	0	Control after treatment	2			7days
M	55	1	22	30	22	0	0	0	0	1	1	1	Control after treatment		2		8days
M	55	0				0	0	0	0	1	0	0	Control after treatment				10days
M	58	0				0	0	0	0	0	0	0	Control after treatment				5days
F	58	0				0	0	0	0	0	0	0	SARS-CoV-2 suspicion	2			10days
M	58	1	16	23	17	0	0	0	0	0	0	0	Control after treatment				9days
M	58	0				0	0	0	0	0	0	0	Control after treatment				7days
F	59	1	26	31	25	0	0	0	0	0	0	0	Control after treatment	2		1	10days
F	59	1	31	32	30	0	0	0	0	0	0	0	Control after treatment			2	5days
F	59	1	32		32	0	0	0	0	1	1	1	Control after treatment	3			5days
F	59	1	32	35	33	0	0	0	0	0	0	0	Control after treatment				8days
M	60	1	14	16	15	0	0	0	0	0	0	0	Control after treatment				8days
F	60	0				0	0	0	0	0	0	0	Control after treatment		2	1	15days
F	62	0				0	0	0	0	0	0	0	Control after treatment				15days
F	63	0				0	0	0	0	0	0	0	Control after treatment				7days
M	63	0				0	0	0	0	0	0	0	Control after treatment				13days
F	68	0				0	0	0	0	0	0	0	SARS-CoV-2 suspicion				11days
F	68	0				0	0	1	0	0	0	0	Control after treatment		1	2	11days
F	85	0				0	0	0	0	0	0	0	Control after treatment				20days
F	89	0				0	0	0	0	0	0	0	Control after treatment				15days

Ct: cycle threshold.

0: negative.

1: positive.

**Table S2:** Cases of reinfection First epidode of infection.

Gender	Age (year)	Ct *RdRp/E*	Ct *N*	Loss of taste and smell	Other symptom	Diarrhea	Cough	Fatigue	Fever	Reasons for the diagnosis	Dose BBIBP-CorV	Dose BNT162b2	Dose ChAdOx1 nCoV-19
F	16	16	17	1	0	0	1	1	0	SARS-CoV-2 suspicion		First dose	
M	22	21	21	0	1	0	0	1	1	Contact case			
F	24	19	20	0	0	1	1	1	1	Contact case		First dose	
F	26	11	9	1	0	0	1	1	1	Contact case	First dose		
M	32	20	21	1	0	0	1	1	1	Contact case			
M	38	15	17	0	0	0	1	1	0	Contact case			
M	43	11	9	0	0	0	1	1	1	Contact case			
M	44	17	20	0	1	0	0	1	1	Contact case			
F	61	17	15	1	0	1	1	1	1	SARS-CoV-2 suspicion			
F	70	21	18	0	1	1	1	1	1	SARS-CoV-2 suspicion			

Second epidode of infection														
Gender	Age (year)	Ct *ORF1ab*	Ct *N*	Ct *E*	Loss of taste and smell	Other symptom	Diarrhea	Cough	Fatigue	Fever	Reasons for the diagnosis	Dose BBIBP-CorV	Dose BNT162b2	Dose ChAdOx1 nCoV-19
F	16	27	21	27	0	1	1	1	1	0	SARS-CoV-2 suspicion		Second dose	
M	22	34		34	0	0	0	0	1	1	Contact case	First dose		
F	24	32	35	30	0	0	0	0	0	0	Contact case		Second dose	
F	26	23	24	20	0	0	0	0	0	0	Contact case	Second dose		
M	32	22	26	22	0	0	0	1	1	1	Contact case			
M	38	21	24	20	0	0	0	1	1	0	Contact case		First dose	
M	43	25	30	24	0	0	0	1	1	1	Contact case			
M	44	23	30	24	0	1	0	0	1	1	Contact case			First dose
F	61	20	25	20	0	0	0	0	0	0	SARS-CoV-2 suspicion			
F	70	19	22	19	0	0	0	1	0	0	SARS-CoV-2 suspicion			

Ct: cycle threshold.

0: negative.

1: positive.

## Figures and Tables

**Table 1:** Odds ratio and adjusted odds ratio for SARS-CoV-2 infection between patients receiving different numbers of vaccine doses.

	OR (95% CI)	P-value	adj. OR (95% CI)	P-value
1 dose vs 2 doses	4.7 (2.14–10.34)	P < 0.01	1.6 (1.3–1,8)	P < 0.01
1 dose vs 3 doses	11.1 (4.94–24.97)	P < 0.01	2.6 (2.1–3.3)	P < 0.01
2 doses vs 3 doses	2.3 (1.64–3.37	P < 0.01	1.6 (1.3–2)	P < 0.01

**Table 2:** General characteristics of patients vaccinated against SARS-CoV-2.

Variables	SARS-CoV-2 positive	SARS-CoV-2 negative	Total	OR (95% CI)	P-value	Adj. OR (95% CI)	P-value
Total	286	310	596				
Gender							
Female	191 (66.8%)	195 (62.9%)	386 (64.8%)	1.2 (0.8–1.6)	0.31	1.1 (0.8–1.4)	0.56
Male	95 (33.2%)	115 (37.1%)	210 (35.2%)				
Vaccinated	286 (53%)	310 (57%)	596 (55.1%)	1.4 (0.9–1.5)	0.3	1.3 (1.1–1.8)	<0.05
Unvaccinated	254 (47%)	232 (42.8%)	486 (44.9%)	Ref.		Ref.	
Total number of patients with 1 dose of vaccine vs unvaccinated	42 (0.1%)	8 (0.02%)	50 (0.1%)	0.20 (0.09–0.45)	<0.01	0.6 (0.5–0.7)	<0.01
1 dose BNT162b2	13 (4.4%)	2 (0.6%)	15 (2.5%)	0.1 (0.03–0.7)	<0.01	2.3 (1.2–4.5)	0.01
1 dose ChAdOx1 nCoV-19	1 (0.3%)	0 (0%)	1 (0.1%)	0.3 (0.01–9)	0.33	1.8 (0.84–4.1)	0.12
1 dose BBIBP-CorV	28 (9.8%)	6 (1.9%)	34 (5.7%)	0.2 (0.1–0.5)	<0.01	2.4 (1.3–4.5)	<0.01
Total number of patients with 2 doses of vaccine vs unvaccinated	176 (0.6%)	158 (0.5%)	334 (0.5%)	0.98 (0.74–1.29)	0.9	1 (0.8–1.1)	0.90
2 doses BNT162b2	26 (9.1%)	32 (10.3%)	58 (9.7%)	1.3 (0.7–2.3)	0.62	1.8 (0.8–4.1)	0.15
2 doses ChAdOx1 nCoV-19	26 (9.1%)	13 (4.2%)	39 (6.5%)	0.5 (0.2–1.1)	<0.01	2.1 (0.7–6.2)	0.17
2 doses BBIBP-CorV	122 (42.6%)	67 (21.6%)	189 (37.7%)	0.6 (0.4–0.8)	<0.01	4.4 (2.1–9.8)	<0,01
1 dose BNT162b2+1 dose ChAdOx1 nCoV-19	0 (0%)	19 (6.1%)	19 (3.2%)	42.6 (2.5–711)	<0.01	20.9 (1.3–323.9)	<0,05
1 dose BNT162b2+1 dose BBIBP-CorV	1 (0.3%)	21 (6.7%)	22 (3.7%)	23 (3.1–172.2)	<0.01	10 (1.7–78)	0.01
1 dose ChAdOx1 nCoV-19 + 1 dose BBIBP-CorV	1 (0.3%)	6 (1.9%)	7 (1.1%)	6.5 (0.7–54.9)	0.06	2 (0.6–22)	0.16
Total number of patients with 3 doses of vaccine vs unvaccinated	68 (0.2%)	144 (0.4%)	212 (0.3%)	2.3 (1.65–3.25)	<0.01	1.6 (1.3–2)	<0.01
3 doses BNT162b2	14 (4.9%)	31 (10%)	45 (7.5%)	2.4 (1.2–4.6)	<0.01	2 (0.9–4.5)	0.75
3 doses ChAdOx1 nCoV-19	10 (3.5%)	20 (6.4%)	30 (5%)	2.2 (1–4.7)	<0.05	2.4 (0.97–6.2)	0.05
3 doses BBIBP-CorV	17 (5.9%)	50 (16.1%)	67 (11.2%)	3.2 (1.8–5.7)	<0.01	3.5 (1.9–6.5)	<0,01
2 doses BNT162b2 +1 dose ChAdOx1 nCoV-19	8 (2.8%)	8 (2.6%)	16 (2.7%)	1.1 (0.4–3)	0.88	1 (0.6–1.7)	0.86
2 doses BNT162b2 +1 dose BBIBP-CorV	1 (0.3%)	2 (0.6%)	3(0.5%)	2.2 (0.2–24.3)	0.58	0.5 (0.3–7.7)	0.6
2 doses ChAdOx1 nCoV-19 + 1 dose BNT162b2	5 (1.7%)	11 (3.5%)	16 (2.7%)	2.4 (0.8–7)	0.17	0.6 (0.8–3.47)	0.2
2 doses ChAdOx1 nCoV-19 + 1 dose BBIBP-CorV	2 (0.7%)	3 (1%)	5 (0.8%)	1.6 (0.2–10)	0.69	1.3 (0.4–3.8)	0.48
2 doses BBIBP-CorV +1 dose BNT162b2	8 (2.8%)	15 (4.8%)	23 (3.8%)	2 (0.8–5)	0.2	1.5 (0.8–2.64)	0.16
2 doses BBIBP-CorV + 1 dose ChAdOx1 nCoV-19	3 (1%)	4 (1.3%)	7 (1.1%)	1.4 (0.3–6.6)	0.73	1.2 (0.5–2.9)	0.65
**Symptoms**							
**Fever**							
Yes	96 (33.6%)	33 (10.7%)	129 (21.6%)	5.3 (3.5–8.2)	<0.01	0.5(0.3–0.7)	<0.01
No	190 (66.4%)	277 (89.3%)	467 (78.4%)				
**Fatigue**							
Yes	175 (61.2%)	108 (34.8%)	283 (47.5%)	2.9 (2.1–4.1)	<0.01	0.6(0.4–0.8)	<0.01
No	111(38.8%)	202 (65.2%)	313 (52.5%)				
**Cough**							
Yes	153 (53.5%)	87 (28%)	240 (40.3%)	2.9 (2–4.1)	<0.01	0.5(0.4–0.5)	<0.01
No	133 (46.5%)	223 (72%)	356 (59.7%)				
**Diarrhea**							
Yes	28 (9.8%)	21 (6.8%)	49 (8.2%)	1.5 (0.8–2.7)	0.18	2.3 (1.3–3.8)	<0.01
No	258 (90.2%)	289 (93.2%)	547 (93.8%)		0.18		
**Loss of taste and/or smell**							
Yes	25 (8.7%)	12 (3.9%)	37 (6.2%)	2.3 (1.1–4.8)	0.01	0.8(0.4–1.5)	0.52
No	261 (91.3%)	298 (96.1%)	559 (93.8%)		0.01		
**Others symptoms**							
Yes	55 (19.2%)	30 (9.7%)	85 (14.3%)	2.2 (1.3–3.5)	<0.01	0.8(0.5–1.2)	0.28
No	231 (80.8%)	280 (90.3%)	511 (85.7%)				
**Categories of age groups**							
≤18 years	20 (6.9%)	31 (10%)	51 (8.5%)	Ref.		Ref.	
19–35 years	94 (32.8%)	114 (36.7%)	208(34.9%)	0.7 (0.4–1.4)	0.31	1(0.9–1.1)	0.05
36–55 years	119 (41.6%)	107 (34.5%)	226 (37.9%)	0.6 (0.3–1.1)	0.07	1(0.9–1.03)	0.9
≥56 years	53 (18.5%)	58 (18.7%)	111 (18.6%)	0.7 (0.3–1.4)	0.95	0.97(0.94–1))	0.5

**Table 3:** The clinical and molecular characteristics of patients reinfected with SARS-CoV-2 and the vaccination status of patients.

Patients	Patient 1	Patient 2	Patient 3	Patient 4	Patient 5	Patient 6	Patient 7	Patient 8	Patient 9	Patient 10
Gender	Female	Male	Female	Female	Male	Male	Male	Male	Female	Female
Age	16 years	22 years	24 years	26 years	32 years	38 years	43 years	44 years	61 years	70 years
Comorbidities	None	None	None	None	None	None	None	None	None	None
**First infection**										
Date of infection	05/04/2021	23/04/2021	24/08/2021	06/08/2021	20/11/2020	27/07/2021	21/12/2020	23/03/2021	03/12/2020	18/11/2020
Clinical presentation	mild infection^a^	mild infection^a^	mild infection	mild infection^a^	mild infection^a^	mild infection^a^	mild infection^a^	mild infection^a^	mild infection^a^	mild infection^a^
**Second infection**										
Date of infection	06/01/2022	10/01/2022	15/01/2022	15/01/2022	05/01/2022	07/01/2022	03/01/2022	10/01/2022	07/01/2022	06/01/2022
Clinical presentation	mild infection^a^	mild infection^a^	Asymptomatic	Asymptomatic	mild infection^a^	mild infection^a^	mild infection^a^	mild infection^a^	Asymptomatic	mild infection^a^
Kit use:	Covsign	Covsign	Covsign	Covsign	Covsign	Covsign	Covsign	Covsign	Covsign	Covsign
**Type of Vaccine and dose**										
BNT162b2	2 doses		2 doses			1 dose				
ChAdOx1 nCoV-19								1 dose		
BBIBP-CorV		1 dose		2 doses						
Unvaccinated					Unvaccinated		Unvaccinated		Unvaccinated	Unvaccinated
Interval days	276 days	262 days	144 days	162 days	411 days	164 days	378 days	293 days	400 days	414 days

a WHO criteria for mild infection.

## Appendix

**Second epidode of infection**.
